# Case report: mechanisms of HIV elite control in two African women

**DOI:** 10.1186/s12879-018-2961-8

**Published:** 2018-01-25

**Authors:** Yumna Moosa, Ramla F. Tanko, Veron Ramsuran, Ravesh Singh, Mashudu Madzivhandila, Nonhlanhla Yende-Zuma, Melissa-Rose Abrahams, Philippe Selhorst, Kamini Gounder, Penny L. Moore, Carolyn Williamson, Salim S. Abdool Karim, Nigel J. Garrett, Wendy A. Burgers

**Affiliations:** 1 Centre for the AIDS Programme of Research in South Africa (CAPRISA), University of KwaZulu-Natal, Durban, South Africa; 2 Institute of Infectious Disease and Molecular Medicine, Faculty of Health Sciences, University of Cape Town, Cape Town, South Africa; 3 School of Laboratory Medicine and Medical Sciences, College of Health Sciences, University of KwaZulu-Natal, Durban, South Africa; 4 Cancer Inflammation Program, Laboratory of Experimental Immunology, Leidos-Frederick, Incorporated, Frederick National Laboratory for Cancer Research, Frederick, USA; 5 Department of Microbiology, National Health Laboratory Services, KZN Academic Complex, Inkosi Albert Luthuli Central Hospital, Durban, South Africa; 6 Centre for HIV and STIs, National Institute for Communicable Diseases (NICD) of the National Health Laboratory Service, Johannesburg, South Africa; 7 HIV Pathogenesis Programme, Doris Duke Medical Research Institute, Nelson R. Mandela School of Medicine, University of KwaZulu-Natal, Durban, South Africa; 8 Faculty of Health Sciences, University of the Witwatersrand, Johannesburg, South Africa; 9 Department of Epidemiology, Mailman School of Public Health, Columbia University, New York, NY USA

**Keywords:** Elite controllers, HIV, Host restriction factors, T-cell responses

## Abstract

**Background:**

The majority of people living with HIV require antiretroviral therapy (ART) for controlling viral replication, however there are rare HIV controllers who spontaneously and durably control HIV in the absence of treatment. Understanding what mediates viral control in these individuals has provided us with insights into the immune mechanisms that may be important to induce for a vaccine or functional cure for HIV. To date, few African elite controllers from high incidence settings have been described. We identified virological controllers from the CAPRISA 002 cohort of HIV-1 subtype C infected women in KwaZulu Natal, South Africa, two (1%) of whom were elite controllers. We examined the genetic, clinical, immunological and virological characteristics of these two elite HIV controllers in detail, to determine whether they exhibit features of putative viral control similar to those described for elite controllers reported in the literature.

**Case presentation:**

In this case report, we present clinical features, CD4^+^ T cell and viral load trajectories for two African women over 7 years of HIV infection. Viral load became undetectable 10 months after HIV infection in Elite Controller 1 (EC1), and after 6 weeks in Elite Controller 2 (EC2), and remained undetectable for the duration of follow-up, in the absence of ART. Both elite controllers expressed multiple HLA Class I and II haplotypes previously associated with slower disease progression (HLA-A*74:01, HLA-B*44:03, HLA-B*81:01, HLA-B*57:03, HLA-DRB1*13). Fitness assays revealed that both women were infected with replication competent viruses, and both expressed higher mRNA levels of *p21*, a host restriction factor associated with viral control. HIV-specific T cell responses were examined using flow cytometry. EC1 mounted high frequency HIV-specific CD8+ T cell responses, including a B*81:01-restricted Gag TL9 response*.* Unusually, EC2 had evidence of pre-infection HIV-specific CD4+ T cell responses.

**Conclusion:**

We identified some features typical of elite controllers, including high magnitude HIV-specific responses and beneficial HLA. In addition, we made the atypical finding of pre-infection HIV-specific immunity in one elite controller, that may have contributed to very early viral control. This report highlights the importance of studying HIV controllers in high incidence settings.

**Electronic supplementary material:**

The online version of this article (10.1186/s12879-018-2961-8) contains supplementary material, which is available to authorized users.

## Background

There is substantial variability in HIV control and disease progression among people living with HIV. Individuals can either resist infection despite repeated HIV exposure, maintain low levels of virus without antiretroviral therapy (ART) (HIV controllers), or control HIV replication to an undetectable level [elite controllers (ECs)] [1]. Understanding the mechanisms that mediate control in these individuals may assist with developing an effective HIV vaccine or cure. However, comprehensive studies on ECs from developing countries with high disease burden are lacking, and since the introduction of universal treatment, opportunities to detect and study these unique individuals are diminishing.

Elite controllers serve as a model for determining factors associated with the protection against HIV. Clinical, immunological, host genetic and virological characteristics have been explored [1] to determine whether elite control is due to a lack of HIV infection of CD4 target cells, replication-defective HIV variants, effective viral control by the host immune system, and/or reduced inflammation with a smaller pool of susceptible CD4 cells [2]. Studies on ECs show that viral control is not solely due to deficient virus, but rather due to host immune responses controlling HIV replication [1–3].

We investigated a longitudinal cohort of South African women who were HIV-uninfected, HIV controllers or HIV progressors. Two ECs were identified and examined in detail for clinical, immunological, host genetic and virological characteristics to understand the mechanisms responsible for viral suppression.

## Case presentation

### Methods

#### Clinical

Women seroconverting in the Centre of the AIDS Programme of Research in South Africa (CAPRISA) HIV prevention studies since 2004, were enrolled into the CAPRISA 002 acute infection cohort study [4, 5]. Controllers were identified and classified either as viraemic or elite, according to their plasma viral load (VL) in the absence of ART [6]. Briefly, viraemic controllers were defined as those with sustained measurements of 50–2000 RNA copies/ml after six months of infection, while elite controllers were those with consecutive undetectable HIV RNA measurements for six months or more. The two elite controllers described in this study had participated in the CAPRISA 004 tenofovir microbicide gel trial [7]. This trial took place between May 2007 and March 2010, and both participants were randomized to the placebo arm. After enrollment into CAPRISA 004, participants had monthly follow-up visits for up to 24 months. PBMC were stored at pre-selected time-points (3, 12 and 24 months). Two HIV rapid antibody tests were performed at monthly visits. Stored plasma, available from prior study visits, was tested by means of RNA PCR, so as to identify the window period for HIV infection and calculate the estimated date of infection. This was defined as two weeks prior to an RNA PCR–positive result if rapid HIV antibody test–negative on the same visit, or as the mid-point between the last HIV negative and first HIV positive antibody test, if no stored plasma was available.

#### Cellular immunology

HIV-specific T-cell responses within peripheral blood mononuclear cell (PBMC) were measured using pools of overlapping peptides spanning Gag, Pol, and Nef of HIV-1 subtype C, as described [8]. IFN-γ ELISPOT assays were used to map epitopes that were predicted from study participants’ human leukocyte antigen (HLA) profiles, as described [8]. T-cell activation and HIV-specific cytokine responses were measured at time-points pre- and post-infection using flow cytometry. The T-cell activation panel included a viability dye (Vivid) and antibodies to detect CD3, CD4, CD8, HLA-DR and CD38. For the evaluation of HIV-specific responses, cells were stained with Vivid, surface-stained with anti-CD4, CD8, CD14 and CD19 (the latter two being exclusion markers), followed by intracellular staining with CD3, IFN-γ, TNF-α and IL-2**.** Samples were acquired on a BD Fortessa and analysed using FlowJo (TreeStar). Cells were gated on singlets, followed by live CD3+ lymphocytes, and then CD4+ or CD8+ subsets. A positive cytokine response was defined as at least twice the background unstimulated sample. Cytokine responses are presented as the background-subtracted total response, representing the frequency of CD4+ or CD8+ T cells producing any of the three cytokines, with the exception of TNF-α-single positive cells, that were excluded due to high TNF-α background responses.

#### Antibody neutralization

Plasma neutralization breadth was determined at 2, 3 and 4 years post-infection for each EC against a panel of 18 heterologous viruses, including 6 subtype C, 6 subtype A and 6 subtype C viruses. The JC53bl-13 (TZM-bl) and 293 T cell lines were obtained through the NIH AIDS Reagent Program, Division of AIDS, NIAID, NIH from Dr. John C. Kappes, Dr. Xiaoyun Wu and Tranzyme Inc. and Dr. Andrew Rice respectively. Both cell lines were cultured in DMEM containing 10% heat-inactivated FBS and 50 μg/ml gentamicin. Cell monolayers were disrupted at confluency by treatment with 0.25% trypsin in 1 mM EDTA. Env-pseudotyped viruses were obtained by co-transfecting Env plasmids with pSG3ΔEnv [9] using FuGENE transfection reagent (Roche) as previously described [10]. Neutralization was measured as described by a reduction in luciferase gene expression after single-round infection of JC53bl-13 cells with Env-pseudotyped viruses [10]. Titers were calculated as the reciprocal plasma dilution (ID_50_) causing 50% reduction of relative light units (RLU).

#### Host genetics

HLA and other host genetic factors associated with viral control in published studies were examined, namely C-C chemokine receptor type 5 *(CCR5)* and cyclin-dependent kinase inhibitor *(CDKN1A-p21)* genes. HLA typing was performed as described [11]. mRNA expression levels of *CCR5* and *p21* were measured using real-time PCR (Roche LightCycler 480 v1.5), with *GAPDH* as a housekeeping control.

#### Virology

*Gag* and *nef* sequences from acute infection were generated from plasma RNA, as described [12], and compared to the subtype-C 2004 consensus (www.hiv.lanl.gov) using EpitopeMatcher. Primary isolate and Gag-Pro-mediated replication capacity was determined, as described [13]. *Gag* DNA copy number was quantified using digital droplet PCR (ddPCR) [14].

### Results

#### Clinical

We studied 233 women, median age 25 years at enrollment, contributing 906 person-years of ART-naïve follow-up (median 3.8 years). Ten women (4.3%) were identified as viraemic controllers, and two (1%) as ECs (EC1 and EC2). Controllers were followed for a median of 6.6 years post-infection (ART-naïve for a median 5.4 years).

**Fig. 1:**
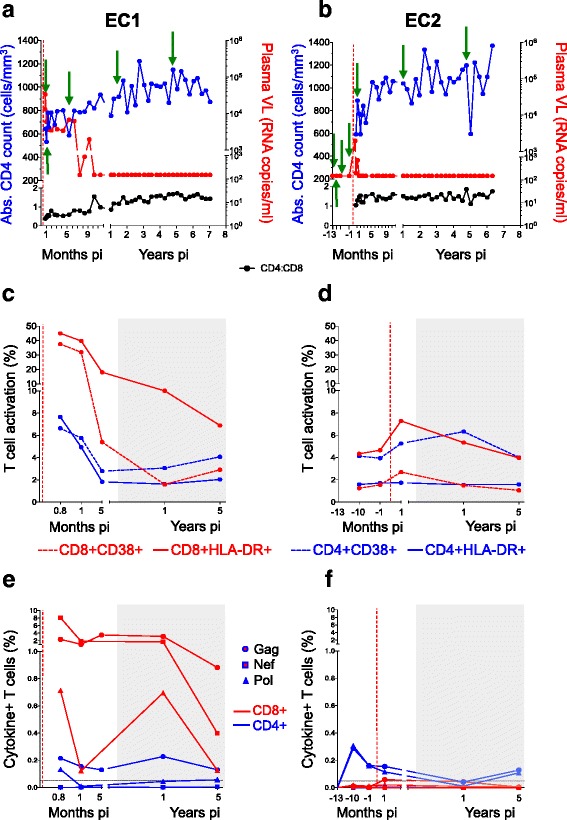
Characterization of HIV elite controllers (ECs). Clinical profiles are presented for EC1 (**a**) and EC2 (**b**) over 7 years of HIV infection. Longitudinal measurements of CD4, viral load (VL) and CD4:CD8 ratio are represented as blue (left axis), red (right axis) and black, respectively. Green arrows indicate time-points selected for immune assays. T-cell activation profiles for EC1 (**c**) and EC2 (**d**) are shown for CD4+ and CD8+ T-cells. CD8+ T-cell activation is indicated in red and CD4+ T-cell activation in blue. Dashed lines indicate the frequency of CD38 expression and solid lines HLA-DR expression for each subset. HIV-specific T-cell responses for EC1 (**e**) and EC2 (**f**) represented as intracellular cytokine responses (IFN-γ, TNF-α and IL-2) to Gag (circle), Nef (square) and Pol (triangle) are indicated (background subtracted). CD4 (blue lines) and CD8 (red lines) responses are indicated. The horizontal black dashed line indicates the cut-off for a positive response (0.05% of T-cells). The grey shaded area on the graphs indicates where VL was lower than the detectable limit of the assay. The red vertical dashed line in all graphs indicates the estimated time of HIV infection

EC1, a 28-year-old woman, presented with a past medical history of recurrent urinary tract infections, tonsillitis, rashes and sexually transmitted infections. Peak VL was 12,902 copies/ml at 23 days post-infection, then dropped to an undetectable level by 10 months and remained suppressed for over 7 years without ART (Fig. 1a). The CD4 count and CD4:CD8 ratio steadily increased throughout follow-up.

EC2, a 38-year-old woman, presented with a past medical history of hypertension and chronic pelvic pain following a caesarean section. Peak VL was 1954 copies/ml at 14 days post-infection (Fig. 1b), became undetectable by 6 weeks and remained suppressed for over 6 years, without ART. The CD4 count steadily increased throughout follow-up, while the CD4:CD8 ratio remained above one.

#### Immunology

T-cell activation levels for EC1 were highest at peak viraemia, declined by 5 months post-infection, and remained low during viral suppression (Fig. 1c). The proportion of activated CD8+ T-cells was higher than CD4+ T-cells. EC2 had considerably lower levels of T-cell activation, and peak CD8+ activation levels also coincided with peak VL, declining during viral suppression (Fig. 1d). T-cell activation for these two ECs was as low as measured for 23 HIV-uninfected individuals rather than for 18 HIV progressors at 5 years post-infection (CD4: 1.8% vs 1.5% and 6.8%; CD8: 5.4% vs 3.7% and 22.7%, respectively) (Additional file 1: Figure S1).

EC1 elicited high magnitude CD8+ T-cell responses secreting IFN-γ, TNF-α and IL-2 against Gag and Nef, with moderate responses to Pol over the first year of infection (Fig. 1e). Lower magnitude CD4+ T-cell responses were mounted to Gag and Pol. T-cell responses declined over 5 years of viral suppression, but remained detectable. Using the IFN-γ ELISPOT assay, the Gag response was mapped to the HLA-B*81:01-restricted TL9 epitope.

EC2 elicited a weaker CD8+ Gag response at 1 year post-infection, that declined over the follow-up period, and low magnitude CD4+ T-cell responses to Gag and Pol were detected at 1 and 5 years post-infection (Fig. 1f). Intriguingly, CD4+ T-cell responses to Gag and Pol were detected at several time points in the year pre-infection, and confirmed in a repeat assay (Additional file 1: Figure S2). However, these responses were absent at 13 months pre-infection. We found no evidence of detectable virus at any of the pre-infection time points using a sensitive quantitative ddPCR assay.

We also investigated plasma neutralization activity against subtype C, B and A viruses in the two ECs at 2, 3 and 4 years post-infection (Additional file 1: Figure S3). EC1 developed neutralizing antibodies to subtype C and A viruses by 2 years post-infection, which waned by 4 years. In contrast, for EC2, no neutralizing antibodies were detected at years 2 and 3 post-infection, while weak neutralization activity to several viruses emerged at year 4.

#### Host genetics

HLA alleles previously shown to associate with slower disease progression were identified within the ECs (Additional file 2: Table S1) [15]. EC1 possessed protective alleles HLA-B*44:03, HLA-B*81:01 and HLA-DRB1*13, while EC2 expressed HLA-A*74:01, HLA-B*57:03 and HLA-DRB1*13.

Higher mRNA expression of the cell-intrinsic inhibitor of HIV reverse transcription, *p21*, was observed among 10 controllers compared to 30 HIV-uninfected participants (median relative ratio 21.8 vs. 13.7, *p* = 0.03, Additional file 1: Figure S4A) [16], with ECs expressing amongst the highest levels. For *CCR5* expression, controllers had a trend towards lower expression compared to progressors (median relative ratio 12.7 vs. 7.6, *p* = 0.06) (Additional file 1: Figure S4B) [17].

#### Virology

Viral sequencing of EC1 at 25 days post-infection showed no evidence of HLA polymorphisms within B*81:01-targeted viral epitopes when compared to a subtype-C consensus sequence. B*44:03-targeted epitopes contained three mismatches, two in Gag and one in Nef. The replication capacity of the EC1 isolate was comparable to the median of 34 primary isolates from the same cohort (data not shown).

For EC2, polymorphisms in predicted epitopes for B*57:03 and B*14:01 alleles were examined at 21 days post-infection. B*57:03-targeted epitopes showed two Gag polymorphisms associated with immune escape, namely the IW9 (A146P polymorphism) and TW10 epitope (T242 N variant). However, the Gag-Pro-mediated replication capacity of EC2 recombinant virus was not impaired and comparable to the median of 71 Gag-Pro-NL4–3 recombinants from the same cohort (data not shown).

## Discussion and conclusions

In this cohort of HIV-1 subtype-C infected South African women, the prevalence of controllers was consistent with 28 cohorts across Europe, Canada, Australia and sub-Saharan Africa [18]. Our findings confirm that elite control is a heterogeneous phenotype with multifactorial mechanisms [1, 6, 18]. The two ECs identified had detectable viremia within 10 months of infection, but were able to control the virus thereafter. Furthermore, they showed low levels of T-cell activation, specific cytokine T-cell responses to HIV antigens, and no evidence for reduced viral replication capacity. Host restriction factors examined here were consistent with previous studies, where ECs expressed higher levels of *p21* compared to HIV-uninfected donors, and lower levels of *CCR5* compared to HIV progressors.

Our results suggest that one mechanism responsible for elite control is a potent HIV-specific host response [2]. For one EC, high magnitude CD8+ responses, including an immunodominant B*81:01 Gag response, shown to be beneficial [15], likely contributed to viral control. In contrast, the other EC had detectable CD4+ responses to Gag and Pol pre-infection. We were unable to detect any evidence of virus in plasma at the ‘pre-infection’ time points, using an ultrasensitive assay. HIV-specific responses in the absence of sustained viral infection could have resulted from either abortive infection, or contained local infection foci that did not spread beyond the site of transmission, and represent ‘immune footprints’ of HIV exposure. HIV-specific responses in the absence of HIV infection have been described in HIV-exposed, but uninfected participants in the iPrEx pre-exposure prophylaxis trial, some of whom mounted high magnitude HIV-specific CD4+ and CD8+ responses [19]. Although the iPrEx study did not determine whether these responses modified the disease course in HIV seroconvertors, the presence of certain HIV-specific responses associated with reduced HIV infection risk [20]. It is possible that pre-infection CD4+ T cell responses in EC2 could have restricted viral replication and contributed to spontaneous control, leading to her elite controller phenotype. Additional studies would be required to investigate this further. Given the potentially high levels of HIV exposure in high incidence settings, it may be interesting to determine whether HIV-specific responses in the absence of HIV infection are more widespread, and could impact HIV infection risk or disease course.

We found heterogenous HIV neutralization activity in the two elite controllers, with neutralizing antibodies waning by year 4 in EC1, and weak responses emerging only at year 4 in EC2. It is thus unlikely that these responses contributed to HIV control. This is consistent with studies demonstrating lower neutralizing antibody titers in ECs compared to viremic patients [21, 22]. In contrast, a recent report described multiple potent broadly neutralizing antibodies detected in an elite controller after more than 20 years of HIV infection [23].

Upregulation of *p21* in T-cells within ECs compared to HIV-uninfected or progressors has been observed previously [16]. *p21* mediates control by blocking cyclin-dependent kinases, a group of host molecules supporting different HIV-1 replication steps [16]. The mechanism of action for the *CCR5* gene has been well-described, i.e. elevated levels associate with loss of control by providing increased co-receptors to the virus [17]. Our study was limited in that we did not test the full breadth of host factors that could potentially have influenced the viral control in the ECs we studied. The ligand for CCR5, CCL3, has been implicated in HIV disease progression [24]. There is also evidence of polymorphisms in APOBEC3G and SAMHD1 contributing to control in ECs [25, 26]. Further studies examining these and other restriction factors may shed more light on the elite controller phenotype.

In conclusion, in this case report we provide a detailed description of two elite controllers from a high burden HIV setting in South Africa, who displayed both typical and atypical features of elite control. Our results highlight the importance of studying HIV control in high incidence settings, where new insights may be gained into mechanisms of durable viral control.

## Additional files

Additional file 1: Figure S1-4. **Figure S1.** T cell activation in the CAPRISA cohorts. CD4+ and CD8+ T cell activation, as measured by the frequency of HLA-DR-positive cells. **Figure S2.** Flow cytometry plots of HIV-specific CD4+ responses in elite controller 2 pre- and post-infection. **Figure S3.** HIV neutralizing antibody responses in elite controllers. **Figure S4.** Host genetic factor expression in the CAPRISA cohorts. (PDF 634 kb)

Additional file 2: Table S1. HLA class I and II alleles identified within the elite controllers. High resolution HLA typing was performed on EC1 and EC2 and is presented in the table. (DOCX 14 kb)

## Abbreviations

IFN-γ: Interferon-gamma
ART: Antiretroviral therapy
CAPRISA: Centre of the AIDS Programme of Research in South Africa
CCR5: C-C chemokine receptor type 5
CDKN1A-p21: Cyclin-dependent kinase inhibitor
ddPCR: Digital droplet PCR
EC: Elite controller
ELISPOT: Enzyme-linked immunosorbent spot
HIV: Human immunodeficency virus
HLA: Human leukocyte antigen
IL-2: Interleukin-2
MRNA: Messenger RNA
PBMC: Peripheral blood mononuclear cells
PCR: Polymerase chain reaction
TNF-α: tumour necrosis factor alpha
VL: Viral load
