# Supplementary material for: Case report: mechanisms of HIV elite control in two African women
Source: BMC Infect Dis. 2018 Jan 25;18:54. doi: 10.1186/s12879-018-2961-8 (PMC5785875; doi:10.1186/s12879-018-2961-8)
Supplement: Supplementary file 1 — Figure S1. T cell activation in the CAPRISA cohorts. CD4+ and CD8+ T cell activation, as measured by the frequency of HLA-DR-positive cells. Figure S2. Flow cytometry plots of HIV-specific CD4+ responses in elite controller 2 pre- and post-infection. Figure S3. HIV neutralizing antibody responses in elite controllers. Figure S4. Host genetic factor expression in the CAPRISA cohorts. (PDF 634 kb) [file 12879_2018_2961_MOESM1_ESM.pdf]

Figure S1

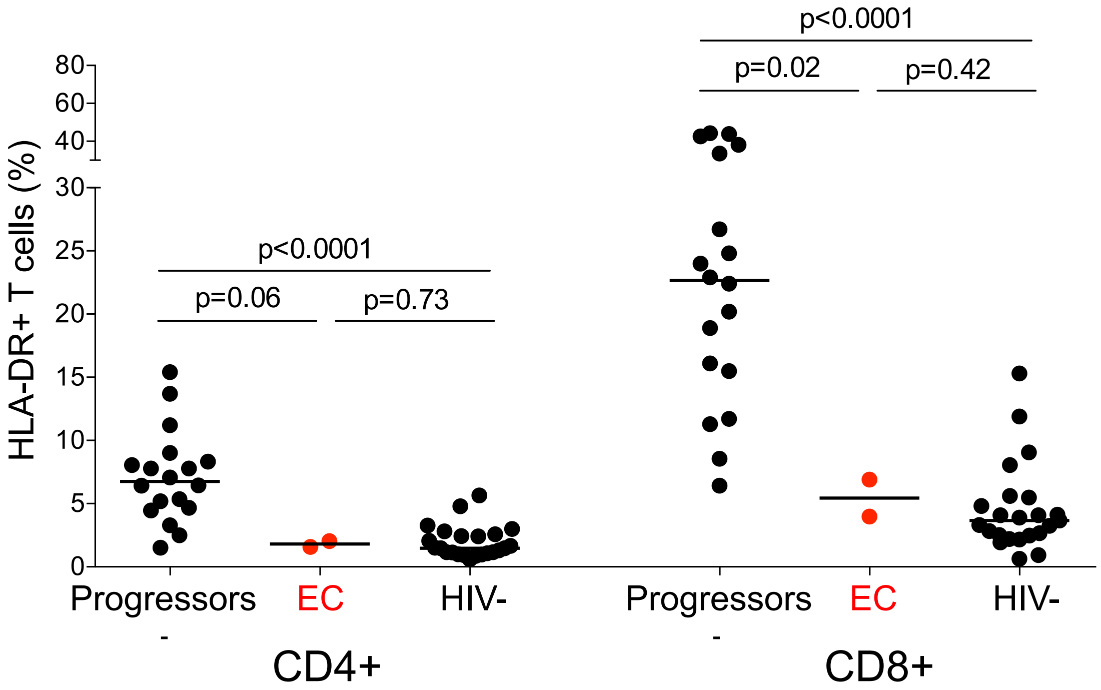

Figure S1. T cell activation in the CAPRISA cohorts. CD4+ and CD8+ T cell activation, as measured by the frequency of HLA-DR-positive cells. HIV progressors in the chronic stage of infection (n=18), the two elite controllers (red dots) and HIV-uninfected individuals (n=24) are shown. T cell activation was measured in elite controllers 5 years post HIV infection. Horizontal lines indicate medians. Statistical comparisons were performed using the Mann-Whitney test.

Figure S2

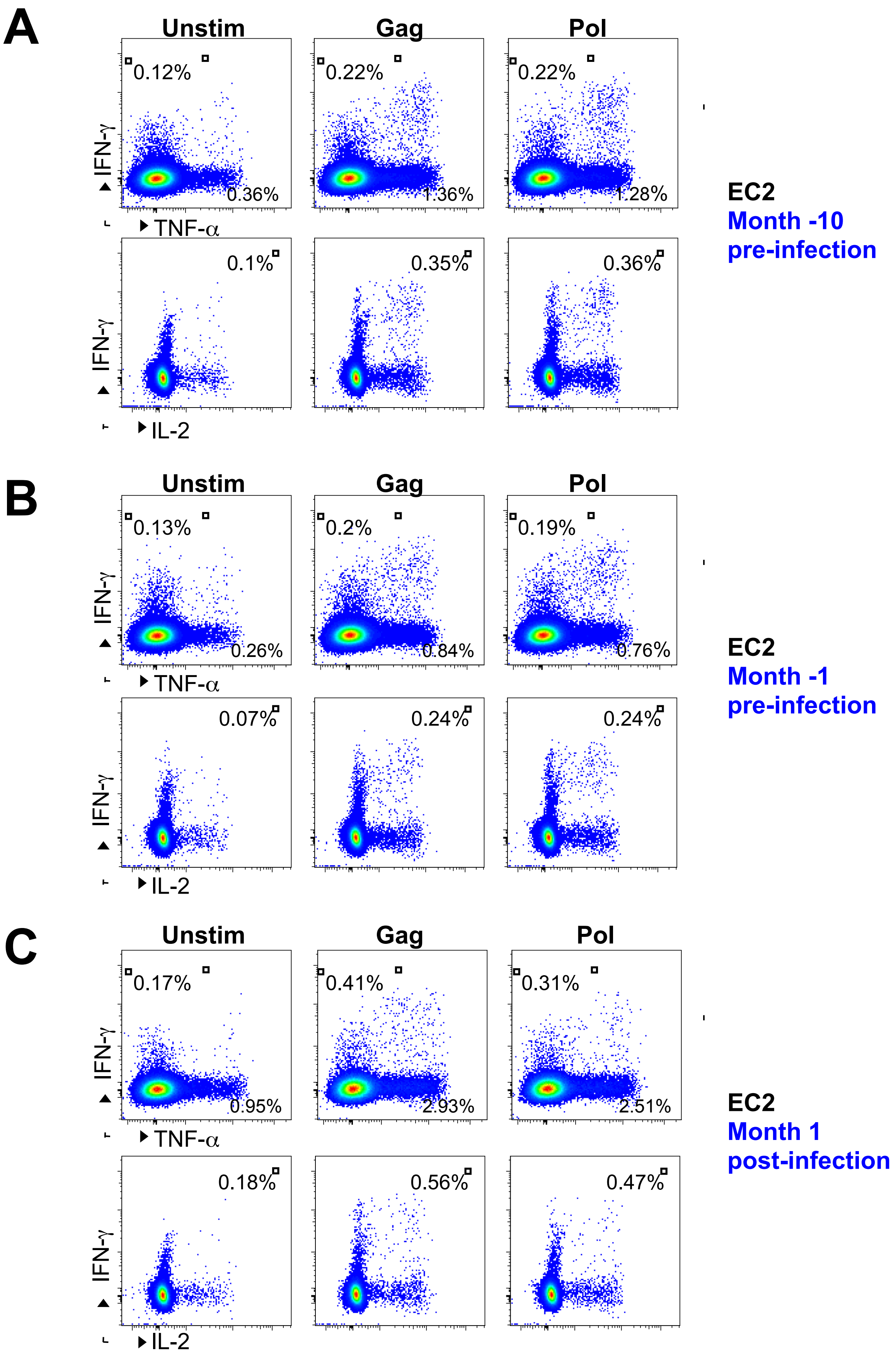
A
Unstim
Gag
Pol
0.12%
0.36%
0.22%
1.36%
0.22%
1.28%
IFN-γ
TNF-α
0.1%
0.35%
0.36%
IFN-γ
IL-2
EC2
Month -10
pre-infection
B
Unstim
Gag
Pol
0.13%
0.26%
0.2%
0.84%
0.19%
0.76%
IFN-γ
TNF-α
0.07%
0.24%
0.24%
IFN-γ
IL-2
EC2
Month -1
pre-infection
C
Unstim
Gag
Pol
0.17%
0.95%
0.41%
2.93%
0.31%
2.51%
IFN-γ
TNF-α
0.18%
0.56%
0.47%
IFN-γ
IL-2
EC2
Month 1
post-infection

Figure S2. Flow cytometry plots of HIV-specific CD4+ responses in elite controller 2 pre- and post-infection. CD4+ T cell IFN-γ, TNF-α and IL-2 cytokine responses from PBMC that were not stimulated (Unstim), or stimulated with Gag or Pol peptides, measured in samples taken 10 months prior to confirmed HIV infection (**A**), 1 month prior to HIV infection (**B**), or 1 month post infection (**C**). Cells were gated on singlets, live CD3+ lymphocytes and then CD4+ cells. The frequency of cytokine positive events is indicated on the plots. A positive cytokine response was defined as at least twice the Unstim sample. Cytokine responses in Figure 1C and F in the manuscript are presented as the background-subtracted total cytokine response, representing the frequency of CD4+ (or CD8+) T cells producing any of the three cytokines. However, due to high TNF-α background responses, TNF-α-single positive cells were excluded from the total response.

Figure S3

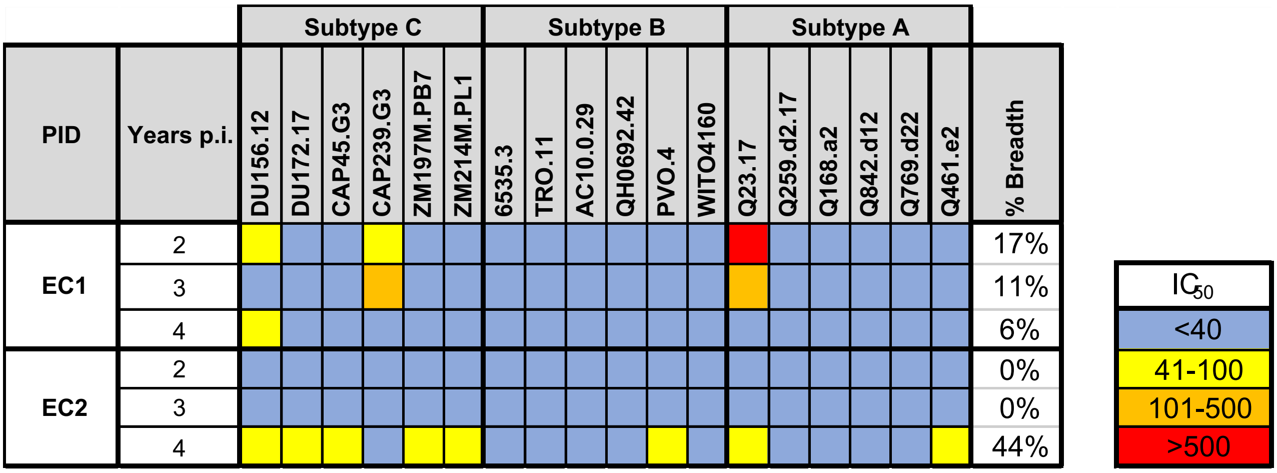

Figure S3. HIV neutralizing antibody responses in elite controllers. Plasma from two, three and four years post-infection (p.i.) was assayed for neutralization breadth against a panel of 18 heterologous viruses, including 6 subtype C, 6 subtype B and 6 subtype A viruses. Neutralization data are represented in a heat-map, with lack of neutralization indicated in blue, IC_{50} titers of 1:41-1:100 in yellow, 1:101-1:500 in orange and greater than 1:500 in red. Percentage breadth is indicated.

Figure S4

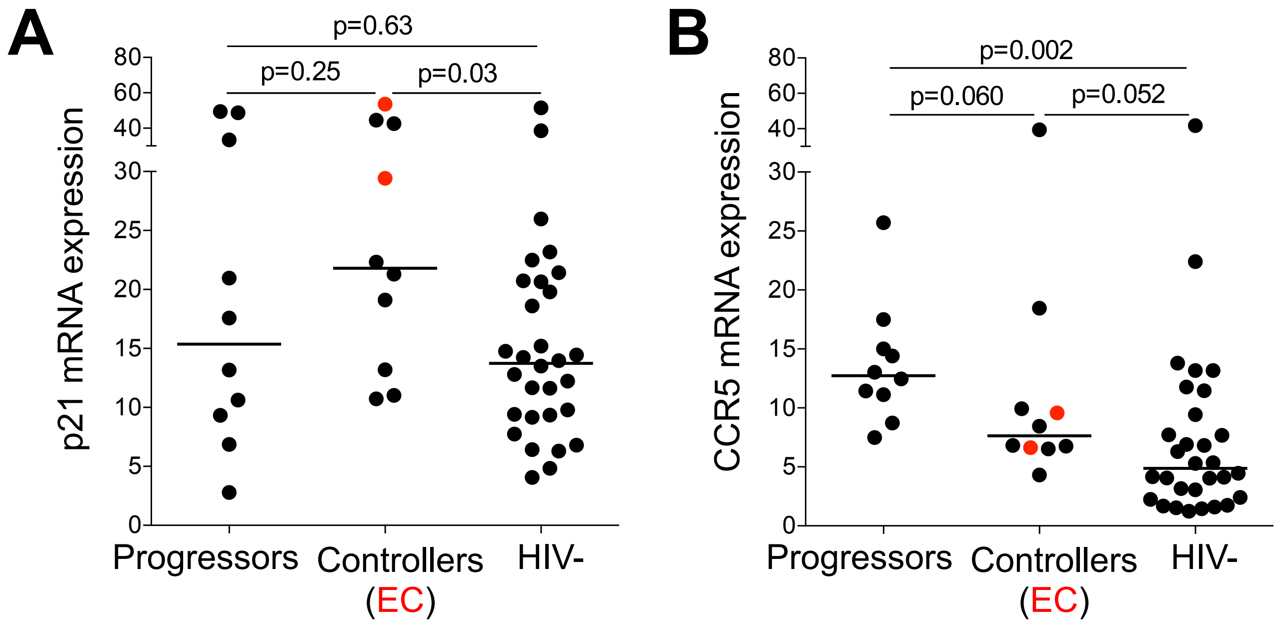

Figure S4. Host genetic factor expression in the CAPRISA cohorts. mRNA expression levels for the p21 (**A**) and CCR5 (**B**) genes were measured in HIV progressors (n=10), HIV controllers (n=10) and HIV-uninfected individuals (n=30). ECs are indicated as red dots. Expression levels are represented relative to the housekeeping gene GAPDH. Horizontal lines indicate medians. Statistical comparisons were performed using the Mann-Whitney test.
