# Supplementary material for: Case report: mechanisms of HIV elite control in two African women
Source: BMC Infect Dis. 2018 Jan 25;18:54. doi: 10.1186/s12879-018-2961-8 (PMC5785875; doi:10.1186/s12879-018-2961-8)
Supplement: Supplementary file 2 — HLA class I and II alleles identified within the elite controllers. High resolution HLA typing was performed on EC1 and EC2 and is presented in the table. (DOCX 14 kb) [file 12879_2018_2961_MOESM2_ESM.docx]

**Additional file 2: HLA class I and II alleles identified within the elite controllers**

| **HLA type** | **EC1** | **EC2** |
| --- | --- | --- |
| HLA-A | A*02:01 | A*68:02 |
|  | A*03:01 | A*74:01 |
| HLA-B | B*44:03 | B*14:01 |
|  | B*81:01 | B*57:03 |
| HLA-C | C*04:01 | C*07:01 |
|  | C*07:01 | C*08:02 |
| HLA-DPB1 | DPB1*04 | DPB1*02 |
|  | DPB1*18 | DPB1*04/06 |
| HLA-DQB1 | DQB1*06 | DQB1*02 |
|  | DQB1*06 | DQB1*02 |
| HLA-DRB1 | DRB1*13 | DRB1*07 |
|  | DRB1*14 | DRB1*13 |
